# Consensus Paper: Latent Autoimmune Cerebellar Ataxia (LACA)

**DOI:** 10.1007/s12311-023-01550-4

**Published:** 2023-03-29

**Authors:** Mario Manto, Marios Hadjivassiliou, José Fidel Baizabal-Carvallo, Christiane S Hampe, Jerome Honnorat, Bastien Joubert, Hiroshi Mitoma, Sergio Muñiz-Castrillo, Aasef G. Shaikh, Alberto Vogrig

**Affiliations:** 1 Service de Neurologie, Médiathèque Jean Jacquy, CHU-Charleroi, Charleroi, Belgium; 2 Service des Neurosciences, University of Mons, Mons, Belgium; 3 Academic Department of Neurosciences, Royal Hallamshire Hospital, Sheffield, UK; 4 Department of Sciences and Engineering, University of Guanajuato, León, Mexico; 5 University of Washington, Seattle, WA USA; 6 French Reference Center on Paraneoplastic Neurological Syndromes, Hospices Civils de Lyon, Hôpital Neurologique, Bron, France; 7 Institut NeuroMyoGene MELIS INSERM U1314/CNRS UMR 5284, Université de Lyon, Université Claude Bernard Lyon 1, Lyon, France; 8 Department of Medical Education, Tokyo Medical University, Tokyo, Japan; 9 Center for Sleep Sciences and Medicine, Stanford University, Palo Alto, CA USA; 10 Louis Stokes Cleveland VA Medical Center, University Hospitals Cleveland Medical Center, Cleveland, OH USA; 11 Clinical Neurology, Udine University Hospital, Azienda Sanitaria Universitaria Friuli Centrale (ASU FC), Udine, Italy; 12 Department of Medicine (DAME), University of Udine, Udine, Italy

**Keywords:** Cerebellum, Immune-mediated cerebellar ataxias, Latent autoimmune cerebellar ataxia, Latent autoimmune diabetes in adults, Cerebellar reserve, Biomarkers

## Abstract

Immune-mediated cerebellar ataxias (IMCAs) have diverse etiologies. Patients with IMCAs develop cerebellar symptoms, characterized mainly by gait ataxia, showing an acute or subacute clinical course. We present a novel concept of latent autoimmune cerebellar ataxia (LACA), analogous to latent autoimmune diabetes in adults (LADA). LADA is a slowly progressive form of autoimmune diabetes where patients are often initially diagnosed with type 2 diabetes. The sole biomarker (serum anti-GAD antibody) is not always present or can fluctuate. However, the disease progresses to pancreatic beta-cell failure and insulin dependency within about 5 years. Due to the unclear autoimmune profile, clinicians often struggle to reach an early diagnosis during the period when insulin production is not severely compromised. LACA is also characterized by a slowly progressive course, lack of obvious autoimmune background, and difficulties in reaching a diagnosis in the absence of clear markers for IMCAs. The authors discuss two aspects of LACA: (*1*) the *not manifestly evident autoimmunity* and (*2*) the *prodromal stage of IMCA’s* characterized by a period of partial neuronal dysfunction where non-specific symptoms may occur. In order to achieve an early intervention and prevent cell death in the cerebellum, identification of the time-window before irreversible neuronal loss is critical. LACA occurs during this time-window when possible preservation of neural plasticity exists. Efforts should be devoted to the early identification of biological, neurophysiological, neuropsychological, morphological (brain morphometry), and multimodal biomarkers allowing early diagnosis and therapeutic intervention and to avoid irreversible neuronal loss.

## Introduction

Immune-mediated cerebellar ataxias (IMCAs) have diverse etiologies [1–6] (Table 1). IMCAs are divided into two groups: (*1*) those in which the trigger of autoimmunity leading to cerebellar damage are known, such as infection (e.g., post-infectious cerebellar syndrome, PiCS, post-infectious cerebellitis), neoplasm (e.g., paraneoplastic cerebellar degeneration, PCD), and gluten sensitivity (gluten ataxia, GA); and (*2*) those with no clear triggers but with serological markers strongly suggestive of IMCAs (e.g., anti-GAD ataxia). When immune-mediated mechanisms leading to cerebellar damage are strongly suspected, but a serological profile does not match any of the known etiologies, the patients are categorized as having primary autoimmune cerebellar ataxia (PACA) [7].

**Table 1:** List of diverse etiologies in immune-mediated cerebellar ataxias (IMCAs)

**1 The etiologies in which cerebellar ataxia is the predominant (isolated or main) clinical phenotype**	
**1.1 Well-established independent etiologies** Common clinical profiles (symptoms, clinical courses, and therapeutic responses) are observed. Mostly, well-characterized autoantibodies are associated. The trigger of the autoimmunity is clear, except anti-GAD ataxia. • Gluten ataxia(gluten sensitivity) • Post-infectious cerebellitis(infection) • Miller Fisher syndrome(infection) • Paraneoplastic cerebellar degeneration(malignancy) • Opsoclonus myoclonus syndrome (infection or malignancy) • Anti-GAD ataxia(unknown)	
**1.2. Clinical spectrum encompassing diverse unknown etiologies** Autoimmunity is suspected, but lack of any specific well-characterized or pathogenic antibodies. • Primary autoimmune cerebellar ataxia (PACA)	
**2 The etiologies in which cerebellar ataxia can be one of various neurological presentations**	
This category encompasses various etiologies characterized by a more global neurological dysfunction where cerebellar ataxia can be one of many neurological features. Mostly, extra-cerebellar symptoms (e.g., seizures, memory deficits, behavioral changes, cognitive changes, sleep disturbances, rigidity, myoclonus, brainstem symptoms, peripheral nerve symptoms, and autonomic dysfunction) are main phenotypes. The prevalence of these etiologies is rare among patients with cerebellar ataxia. • Cerebellar ataxia associated with autoantibodies toward ion channels/related proteins Anti-VGCC, Caspr2, DPPX • Cerebellar ataxia associated with autoantibodies toward synaptic adhesion molecules Anti-LGI1, IgLON5, mGluR delta • Cerebellar ataxia associated with autoantibodies toward transmitter receptors Anti-NMDAR, AMPAR, mGluR1, mGluR2, mGluR5, GABA_A_R, GABA_B_R, GlycineR • Autoimmunities toward myelin-related proteins Anti-MAG • Autoimmunities toward glial cells GFAP astrocytopathy • Perivascular T cell inflammation in the brainstem Chronic lymphocytic inflammation with pontine perivascular enhancement responsive to steroids	

Patients with IMCAs generally develop a cerebellar motor syndrome, characterized mainly by gait ataxia, with an acute or subacute clinical course. Identification of well-characterized antibodies (Abs) is essential in the diagnosis of some IMCAs: for example, onconeural Abs in PCD, anti-gliadin and anti-TG6 Abs in GA, and high-titer of anti-GAD antibodies (anti-GAD Abs) in anti-GAD ataxia [1–6]. In contrast, some patients exhibit ataxia with a slowly progressive time-course, without obvious autoimmune background [8–10]. Neurological symptoms other than ataxia sometimes precede the development of ataxia, suggestive of the existence of *a prodromal stage* in IMCAs [9–11].

The condition is analogous to latent autoimmune diabetes in adults (LADA), which is characterized by an atypical presentation of autoimmune type 1 diabetes mellitus (DM), slow and progressive course, and sometimes fluctuating association of anti-GAD Abs, the sole autoimmune biomarker [12–15]. Patients are initially diagnosed with type 2 DM and gradual decompensation due to islet autoimmunity and insulin deficiency leading to insulin dependency [16]. In analogy with LADA, we propose a novel clinical concept of latent autoimmune cerebellar ataxia (LACA) to underline two conditions: “not manifestly evident autoimmune pathologies” and “prodromal stage of IMCAs.”

This novel clinical concept, LACA, will provide a framework for early intervention during a period when cerebellar reserve, capacities for compensation and restoration, is preserved [17]. The current consensus paper aims to discuss the validity of the LACA hypothesis. From this point of view, we revisit clinical profiles of CA associated with low-titer of anti-GAD Abs CA, GA, and PCD.

## Latent Autoimmune Cerebellar Ataxia (Mario Mano, Marios Hadjivassiliou, Hiroshi Mitoma)

### Slowly Progressive Cerebellar Ataxia and “Not Manifestly Evident” Autoimmunity

There are reports of patients showing slowly progressive ataxia without definite autoimmune triggers or well-characterized Abs responding positively to immunotherapies [8–10]. Some researchers questioned if these patients have IMCAs. They argue that it is uncertain whether their autoimmune tendency is directly or indirectly responsible for the insult to the cerebellum based on the following two problems [18].

The first problem is the significance of the presence of autoantibodies. If such antibodies are not pathogenic, then autoantibodies and ataxia coexist, and the ataxia is caused by metabolic/toxic or degenerative mechanisms other than autoimmunity. It should also be noted that some autoantibodies such as anti-thyroid Abs and low-titer anti-GAD Abs can be found in healthy subjects, albeit at low frequencies [18]. In addition, some antibodies, e.g., anti-NH2-terminal of α-enolase (NAE) Abs, co-exist in patients with multiple system atrophy, suggesting degenerative changes might induce secondarily autoimmune processes [19].

The second problem is the lack of autoimmune features. For example, in diagnosis criteria of PACA, clinical features that suggest autoimmune etiology were proposed, including acute or subacute time course, midline cerebellar atrophy, CSF pleocytosis and/or positive CSF restricted IgG oligoclonal bands, history of other autoimmune disorders, or family history of autoimmune disorders [7]. However, some of these patients may not exhibit many of the autoimmunity-pointing features defined by PACA criteria.

Thus, the reported cases were diagnosed as IMCAs for the first time as a result of response to immunotherapies or intrathecal Ab synthesis [8–10] and the exclusion of other etiologies. Faced with such cases, clinicians may have difficulty making a diagnosis of IMCAs.

### “Prodromal Stage” in Immune-Mediated Cerebellar Ataxias

Degenerative diseases, including Alzheimer’s, idiopathic Parkinson’s disease, progressive supranuclear palsy, usually develop gradually, over the course of months to years. It is well known that some non-neurological and neurological symptoms (e.g., anosmia in Parkinson’s disease) can be present at the prodromal stage [20, 21]. Consistently, a pre-symptomatic or prodromal stage was observed in an animal model of degenerative ataxia [22]. In patients with degenerative ataxia, pre-ataxic symptoms have recently been reported in gait stability [23] and ocular movements [24]. Time-course of development of ataxia in spinocerebellar ataxia (SCA) types 1, 2, 3, and 6 mutation carriers was traced longitudinally and revealed marginal progression in the prodromal period, followed by increasing progression once ataxia is established [25]. On the other hand, it should be acknowledged that particular neurological symptoms such as brainstem attacks preceded the manifestation of ataxia in some patients with IMCAs [9–11]. Some organ-specific autoimmune disorders can also be present as prodromal [9].

### The Analogy with Latent Autoimmune Diabetes in Adults/Slowly Progressive Insulin-Dependent Diabetes Mellitus

Anti-GAD Abs are specifically associated with type 1 DM, characterized by acute onset with an immediate requirement for insulin therapy and immune-mediated destruction of pancreatic beta-cells. Interestingly, anti-GAD Abs are also detected in ~10% of patients with adult-onset DM initially diagnosed as type 2 DM [13–16]. Thyroid and gastric auto-immunity are also frequently associated. These patients, who initially do not require insulin treatment, gradually become insulin-dependent. This atypical subset of autoimmune DM is referred to as slowly progressive insulin-dependent DM (SPIDDM) [26] or latent autoimmune diabetes in adults (LADA) [12, 27]. The presence of anti-GAD Abs reflects the autoimmune-mediated inflammation in the islet.

LADA shows a lack of manifestly evident autoimmunity. In addition to atypical clinical courses (slow progression to insulin dependency), anti-GAD Abs are not repeatedly detected. It was reported that after 3 years of follow-up, 37% of LADA patients remained anti-GAD Ab positive, 20% fluctuated between positivity and negativity, and the remaining 43% became anti-GAD Ab negative [15].

The time course of LADA is now classified into three stages [16]. At the first stage of the disease, genetically triggered autoimmunity slowly destroys the beta-cell and reduces insulin secretion. At a second stage, exposure to an unhealthy lifestyle causes insulin resistance and overload of beta-cells. Eventually, any compensations by beta-cells fail to meet the increasing insulin need, resulting in hyperglycemia and, finally, an insulin-dependent status. In other words, the slowly progressive immune-mediated inflammation ultimately disrupts beta-cells’ reserve capacities [13]. Thus, clinicians often struggle to reach an early diagnosis. Careful estimations on insulin deficiency (e.g., C-peptide test) are recommended in LADA/SPIDDM [13, 14]. Recent studies using ELISA methods show that the titer of anti-GAD Abs has no relevance to insulin-requiring diabetes [14]. The progression to a stage of beta-cell destruction occurs in patients with high-titer and low-titer. Furthermore, an early intervention targeting anti-GAD Ab-positive individuals without manifest diabetes is proposed [16].

In conclusion, a clinical notion of LADA/SPIDDM argues for the importance of early intervention by stressing two critical characteristics: (*1*) autoimmune etiology is *present* but *easily overlooked*, (*2*) beta-cell deficiency is *potential* but not *active /symptomatic* in the early stage (a prodromal stage).

### Definition of Latent Autoimmune Cerebellar Ataxia (LACA)

**Fig. 1:**
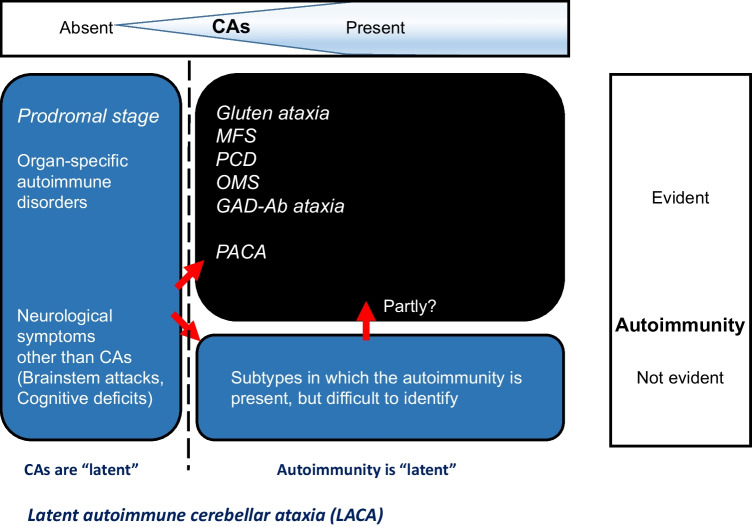
A definition of LACA. Prodromal IMCAs and IMCAs are classified based on the manifestation of two factors: cerebellar ataxias and autoimmunity. LACA can be utilized to describe situations in which cerebellar ataxias are latent or the autoimmunity is latent. IMCAs, immune-mediated cerebellar ataxias; LACA, latent autoimmune cerebellar ataxia; MFS, Miller Fisher syndrome; PCD, paraneoplastic cerebellar degeneration; OMS, opsoclonus myoclonus syndrome; PACA, primary autoimmune cerebellar ataxia

We propose the concept of latent autoimmune cerebellar ataxia (LACA) analogous to LADA. The term LACA should be used in the following situations (see also Fig. 1):

1. An autoimmune etiology is *present* but *not easily detectable* since it is not associated with personal history of autoimmune diseases or well-characterized autoantibodies.
2. The ataxia is *subclinical* or *so mild that is difficult to detect* on clinical examination, and non-specific symptoms or other non-cerebellar neurological manifestations may precede the manifestation of ataxia. This stage can be retrospectively identified as prodromal.
  LACA by definition is likely to follow a course of *slow progression*. Ultimately, the autoimmune mechanisms will affect the cerebellum, resulting in clinical ataxia and eventually marked cerebellar atrophy.
  The notion of LACA is introduced to encourage clinicians to carefully examine the possibility of slow-evolving IMCA, as well as to stress the importance of the early intervention of immunotherapies during a period when there is cerebellar reserve.

**Fig. 2:**
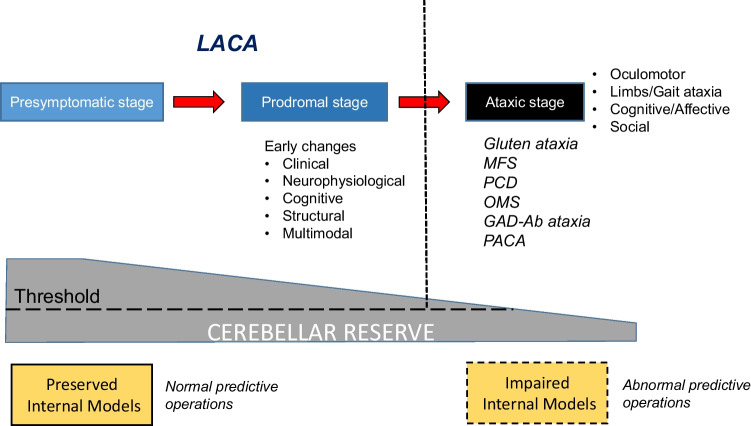
A clinical course from presymptomatic stage and prodromal stage to ataxic stage. LACA can be used to describe the prodromal stage in IMCAs. During a period of the presymptomatic and prodromal stages, the internal model is preserved, leading to normal predictive operations, whereas, in ataxic stage, the internal model is impaired, resulting in a development of cerebellar ataxias. As the disease progresses, cerebellar reserve is lost. IMCAs, immune-mediated cerebellar ataxias; LACA, latent autoimmune cerebellar ataxia; MFS, Miller Fisher syndrome; PCD, paraneoplastic cerebellar degeneration; OMS, opsoclonus myoclonus syndrome; PACA, primary autoimmune cerebellar ataxia

A clinical course is shown in Fig. 2 and the comparisons between LADA and LACA are shown in Table 2.

**Table 2:** Comparisons between latent autoimmune diabetes in adults (LADA) and latent autoimmune cerebellar ataxia (LACA)

	Latent autoimmune diabetes in adults (LADA)	Latent autoimmune cerebellar ataxia (LACA)
Autoimmunity	Present, but *not easily detectable* because often subclinical	
Prodromal stage	• Present • Compensations for islet autoimmunity and insulin deficiency are disrupted, LADA becomes manifest	• Present • Brainstem attacks were reported • A disruption of cerebellar reserve leads to a clinical manifestation of LACA
Clinical course	• Adult onset (30–50 years), slowly progressive to insulin dependency • Anti-GAD antibodies positive • Different from auto-immune diabetes type 1 and ketosis prone diabetes	• Mostly, slowly progressive • Possible detection of circulating antibodies • May evolve into IMCA
Outcome of autoimmune insult	β cell destruction	Cerebellar atrophy
	• β cell destruction and cerebellar atrophy is *potential* but may be *not active/symptomatic* • Early intervention warranted • Possible preventive therapies	

*Abbreviations*: *IMCAs*, immune-mediated cerebellar ataxias

## “Not Manifestly Evident” Autoimmunity Observed in LACA

This section highlights “not manifestly evident” autoimmunity in LACA through examples of CA associated with anti-GAD Abs and paraneoplastic cerebellar degeneration (PCD). In the “Slowly Progressive Cerebellar Ataxia and “Not Manifestly Evident” Autoimmunity” section, we proposed two factors of “not manifestly evident” autoimmunity: (1) the presence of poorly characterized autoantibodies and (2) the lack of autoimmune features. Here we will discuss these two features in CA associated with anti-GAD Abs and PCD, respectively. The significance of poorly characterized Abs associated with cerebellar ataxia needs to be carefully followed in conjunction with the manifestation of other autoimmunity features.

### Anti-GAD Autoantibodies: Titer and Epitope Specificity

#### High-Titer and Low-Titer Anti-GAD Ab and CA (Hiroshi Mitoma, Christiane S Hampe, Marios Hadjivassiliou)

High-titer anti-GAD65 Ab in serum (often also found in the CSF) leads to the diagnosis of anti-GAD ataxia [2, 4]. The titer is usually above10,000 U/mL (or 10- to 100-fold higher compared to those of patients with type 1 DM) [2, 4]. In patients with ataxia and serum anti-GAD Abs exceeding 2000 U/mL, one can safely consider anti-GAD ataxia [6]. In contrast, the significance of low-titer anti-GAD Ab is unclear. However, it should be acknowledged that a portion of patients with low-titer anti-GAD Ab and CA must have immune-mediated etiologies since immunotherapies improved are effective. We will use the terms “high-titer” and “low-titer” as defined in the respective studies rather than reporting specific values due to the use of different anti-GAD Ab assays.

The practical problem is that the autoimmune nature may *not be easily detectable* in CA associated with low-titer anti-GAD Ab [8–10, 28–35]*.* Only half of such patients have a history of autoimmune diseases, and very few showed intrathecal production of anti-GAD Abs [8–10, 28–35]. Moreover, all patients showed pan-cerebellar ataxia with insidious clinical courses [8–10, 28–35]. Due to these clinical presentations atypical for IMCAs, the presence of low-titer anti-GAD Ab does not provide obvious evidence that immune-mediated mechanisms insult the cerebellum. The autoimmune significance of low-titer anti-GAD Ab was determined only after confirming the responsiveness to immunotherapies that relied on these few clues [8–10, 28–35].

Thus, it is likely that the majority of patients with high-titer of anti-GAD Ab, and not, as yet, any clinical evidence of ataxia, may well have LACA, and that a smaller proportion of patients with low-titer anti-GAD Ab may also have LACA.

Notably, the epitope specificities of anti-GAD Abs in type 1 DM differ significantly from those in neurological diseases associated with anti-GAD Ab. In addition, it was suggested that high-titer anti-GAD Ab decreased GABA release, while low-titer anti-GAD Ab had no such pathogenic actions [32]. These characteristics of low-titer anti-GAD Ab and CA show the following principle: “The presence of auto-Abs alone may not support the diagnosis of IMCAs due to multiple epitopes, while low-titer auto-Abs are not necessarily correlated with only low-level insults on the cerebellum.” This principle should be considered when diagnosing “not manifestly evident” autoimmunity. Since epitope specificities have not been considered so far in the diagnosis of neuroimmune diseases, we will discuss methodological problems in defining epitopes in the next section.

#### Importance of Epitope Mapping of Anti-GAD Abs (Christiane S Hampe)

Epitope mapping of disease-specific anti-GAD Abs can support the correct diagnosis and prediction of disease, understanding of the underlying autoimmune response, identification of antibodies with pathologic potential, and development of therapeutics.

**Fig. 3:**
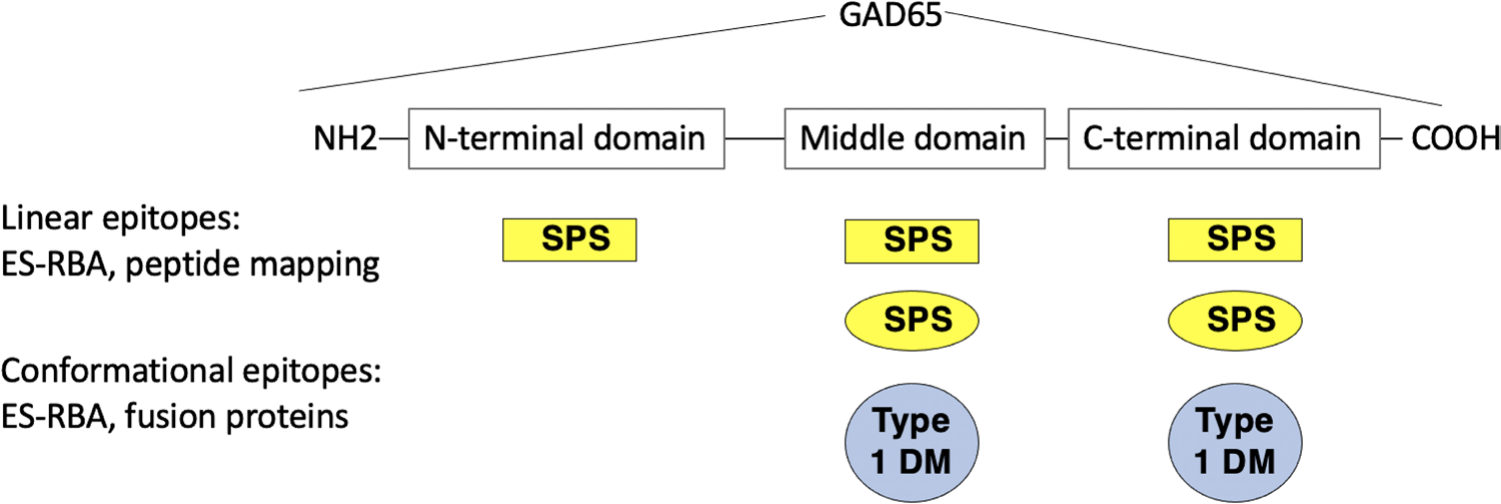
Anti-GAD Ab epitope domains recognized by antibodies in different diseases. Linear anti-GAD Ab epitopes recognized by patients with SPS (rectangular boxes) have been identified using both peptide mapping and ES-RBA. These epitopes are dispersed across the entire GAD molecule. Conformational anti-GAD Ab epitopes recognized by patients with SPS (yellow ovals) or patients with type 1 DM (blue circles) have been identified by the use of GAD65/67 fusion proteins and ES-RBA

Disease-specific anti-GAD Ab epitopes in type 1 DM and neurological disorders, such as Stiff Person Syndrome (SPS), were evident already in the early 90s, when Baekkeskov et al. found that anti-GAD Abs in patients with SPS recognized both linear and conformational GAD epitopes, while those in patients with type 1 DM were dependent on the conformation integrity of the molecule [36]. Subsequent analyses utilized fusion proteins substituting regions of GAD65 with those of the slightly larger isoform GAD67. GAD65/67 fusion proteins [37] helped to identify two major regions in the middle and the C-terminus that contained conformational epitopes relevant to type 1 DM, while anti-GAD Ab in patients with SPS recognized epitopes located also at the N-terminus [38] (Fig. 3). However, there were considerable shortcomings associated with this epitope mapping approach. The use of GAD67 as a “scaffold” for GAD65 epitope regions depends on the relative lack of antigenicity of GAD67. Indeed, antibodies in type 1 DM only occasionally recognize GAD67 [39]. However, anti-GAD Ab in SPS and other neurological disorders frequently react with GAD67, and GAD65/67 fusion proteins are therefore of limited use for epitope analyses, especially in neurological disorders [31].

An epitope mapping assay (epitope-specific radioligand binding assay (ES-RBA)) allows the detection of conformational and linear GAD65-specific antibody epitopes and was able to distinguish between anti-GAD Abs in sera of patients with type 1 DM, SPS, limbic encephalitis, CA, and anti-GAD Ab-positive epilepsy [40, 41] (Fig. 3). This epitope recognition was independent of anti-GAD Ab titer, a crucial aspect as anti-GAD Ab titers often vary considerably. Importantly, other studies using GAD65 fragments for epitope mapping in patients with the same neurological autoimmune diseases could not identify these disease-specific epitopes [42, 43], underlining the relevance of this approach for the identification of disease-specific anti-GAD Ab epitopes.

Therefore, the presence of anti-GAD Abs in itself is not sufficient for a precise diagnosis. Furthermore, low anti-GAD Ab titers do not always suggest a more benign pathogenesis. Other factors, such as anti-GAD Ab epitopes (linear and conformational), location of anti-GAD Ab production (systemic and intrathecal), and IgG subclasses, need to be part of a careful evaluation of the underlying autoimmune response. Future studies to identify anti-GAD Ab epitopes in LACA are needed to establish a disease-specific pattern, which may aide clinicians in the diagnosis and targeted therapy.

### “Not Manifestly Evident” Autoimmunity in PCD (Sergio Muñiz-Castrillo, Alberto Vogrig, Bastien Joubert, Jérôme Honnorat)

Paraneoplastic cerebellar degeneration (PCD), often manifesting as rapidly progressive cerebellar syndrome, is defined as a severe subacute pancerebellar syndrome triggered by cancer [44, 45]. PCD has been reported as one of the most common paraneoplastic neurological syndromes (PNS), accounting for nearly 20–30% of those fulfilling the criteria for definite PNS [46–49].

#### Well and Poorly Characterized Onconeural Antibodies

A characteristic of PCD, as in other PNS, is the association with autoantibodies that target a great diversity of neural antigens (Table 3). Most of these antigens are located at the intracellular level; hence, the antibodies are thought not to be directly involved in the pathogenesis of the disease that is instead mostly mediated by cytotoxic T cells [50]. Nevertheless, most of these antibodies are reliable biomarkers of an underlying cancer and therefore useful to guide the tumor screening [44], as nearly 65% of these patients are not known to have cancer at the time of diagnosis [51, 52]. In two PCD series, the most common anti-neural Abs identified were, in decreasing order of frequency, Yo, Hu, CV2/CRMP5, Tr/DNER (delta/notch-like epidermal growth factor-related receptor), Ri, and Ma2 [51, 52]. Furthermore, the oncological accompaniments considerably differ between these Abs [53–58] (Table 3). Recently, anti-Kelch-like protein 11 (KLHL11) Abs have also been described in association with testicular tumors and brainstem-cerebellar involvement [59].

**Table 3:** Main antibody and cancer associations with PCD

Antibody	Predominant sex, median age (y)	Frequency of cerebellar involvement (%)	Other neurological phenotypes	Usual tumors	Frequency of cancer (%)	Ref.
Yo/PCA-1	>95% F, 60	>90	Brainstem, cranial nerve involvement, or peripheral neuropathy may co-occur with PCD (10%)	Ovary and breast	>90	[62, 77]
Tr/DNER	70% M, 55	>90	Uncommonly LE or co-occurring encephalopathy or optic neuritis	Hodgkin lymphoma	90	[53, 54]
KLHL11	>95% M, 45	85	Co-occurring brainstem involvement is almost constant, LE, myelitis	Testicular	80	[59]
Ri/ANNA-2	70% F, 65	50–70	Brainstem, OMS, movement disorders	Breast > lung	>70	[56, 57, ]
MAP1B/PCA-2	50% M/F, 70	40	Sensorimotor neuropathy, EM	SCLC, NSCLC, breast	80	[117]
Ma2	70% M, 55	20–30	LE, diencephalitis, brainstem encephalitis	Testicular, NSCLC	>75	[58]
CV2/CRMP5	60% M, 60	20–25	EM, SNN, chorea, uveo-retinal involvement	SCLC, malignant thymoma	>80	[118, 119]
SOX-1/AGNA	60% M, 60	20	LEMS	SCLC	>90	[120]
Amphiphysin	60% F, 65	15	Polyradiculoneuropathy, SNN, EM, SPS	SCLC, breast	80	[121]
Hu/ANNA-1	70% M, 65	10–20	SNN, chronic gastrointestinal pseudo-obstruction, EM, LE	SCLC >> NSCLC	85	[55]
P/Q VGCC	>95% M, 65	<2	Co-occurring LEMS (50%)	SCLC	90	[122, 123]

*Abbreviations*: *AGNA*, anti-glial nuclear antibody; *ANNA*, antineuronal nuclear antibody; *CRMP5*, collapsin response-mediator protein-5; *DNER*, delta/notch-like epidermal growth factor-related receptor; *EM*, encephalomyelitis; *F*, female; *KLHL11*, Kelch-like protein 11; *LE*, limbic encephalitis; *LEMS*, Lambert-Eaton myasthenic syndrome; *MAP1B*, microtubule associated protein 1B; *M*, male; *NSCLC*, non-small-cell lung cancer; *OMS*, opsoclonus-myoclonus syndrome; *PCA*, Purkinje cell antibody; *PCD*, paraneoplastic cerebellar degeneration; *SCLC*, small-cell lung cancer; *SNN*, sensory neuronopathy; *SPS*, stiff-person syndrome; *VGCC*, voltage-gated calcium channels; *y*, years

Besides, several additional antibodies have been identified in only a few PCD patients (Table 4), such as Abs directed against TRIM9/67 and metabotropic glutamate receptor 2 [60, 61]; their characterization therefore warrants further investigation. Finally, seronegative cases may account for approximately 20% of PCD, being associated with gynecological cancers, lymphomas (non-Hodgkin and Hodgkin), and lung cancer in women, and with lung, Hodgkin lymphoma, and genitourinary cancers in men [52]. Conversely, cancer is not found in about 10% of Ab-positive suspected PCD [51, 52].

**Table 4:** Poorly characterized antibodies associated with PCD

Antibody	Number of cases reported	Frequency of cerebellar involvement (%)	Other neurological phenotypes	Associated tumors	Frequency of cancer (%)	Ref.
TRIM9/67	2	100	-	NSCLC	100	[60]
mGluR2	2	100	-	Neuroendocrine cancer, alveolar rhabdomyosarcoma	100	[61]
Protein kinase Cγ	2	100	-	NSCLC, hepatic adenocarcinoma	100	[124, 125]
CARP VIII	3	100	-	Melanoma, ovarian and breast cancer	100	[126–128]
ARHGAP26	10	70	Cognitive impairment, hyperekplexia	Diverse carcinomas, B-cell lymphoma, melanoma	50	[129]
Neuronal intermediate filament (light chain)	21	50	Encephalopathy, myelopathy	Neuroendocrine carcinomas	80	[130]
ITPR1	25	50	Peripheral neuropathy, encephalitis, myelopathy	Breast and others carcinomas	30	[131, 132]
ANNA-3	11	30	Sensorimotor neuropathy, LE, myelopathy	SCLC	80	[133]

*Abbreviations*: *ANNA-3*, anti-nuclear antibody 3; *ARHGAP26*, Rho GTPase-activating protein 26; *CARP VIII*, carbonic anhydrase-related protein VIII; *ITPR1*, inositol 1,4,5-triphosphate receptor 1; *LE*, limbic encephalitis; *mGluR2*, metabotropic glutamate receptor 2; *NSCLC*, non-small-cell lung cancer; *PCD*, paraneoplastic cerebellar degeneration; *SCLC*, small-cell lung cancer; *TRIM*, tripartite motif-containing protein

#### Main Clinical Features and Temporal Patterns

PCD commonly presents as a truncal and appendicular ataxia developed over a matter of weeks [51]. Additionally, horizontal nystagmus is an almost constant feature, often with some vertical and torsional component, whereas down-beat nystagmus in particular has been proposed as a hallmark of PCD [55, 62]. Hyperacute presentations in less than 24 h have been described in patients with anti-Yo and anti-Hu Abs, though they only account for approximately 5% of all PCDs [56, 62–64]. Less commonly, the cerebellar presentation can be slowly progressive over months, especially in patients with anti-Ri and anti-Ma2 Abs [57, 65].

## LACA: the Prodromal Stage

There has been cumulating evidence suggesting that some neurological symptoms precede the manifestation of ataxia in degenerative/genetic ataxias. Alteration of gait and postural sway control was detected in subjects with prodromal SCA2 using a wearable sensor-based system [23]. Pre-ataxic changes in SCA3 patients were reported in vestibulo-ocular reflex gain, main sequence of vertical volitional saccades, and slow-phase velocity of central and gaze-evoked (SPV-GE) nystagmus in SCA3 [24]. We argue the existence of a “prodromal stage” that precedes the manifestation of CAs also in IMCAs. We retrospectively review prodromal symptoms in low-titer anti-GAD ataxia (section “Prodromal Stage in Patients with Anti-GAD Antibodies (José Fidel Baizabal-Carvallo)”), PCD (section “Prodromal Stage in PCD (Sergio Muñiz-Castrillo, Alberto Vogrig, Bastien Joubert, Jérôme Honnorat)”), gluten ataxia (GA) (section “Prodromal Stage in Gluten Ataxia (Marios Hadjivassiliou)”), and post-infectious cerebellar syndrome (section “Prodromal Stage in Post-infectious Cerebellar Syndrome (PiCS) (José Fidel Baizabal-Carvallo)”).

### Prodromal Stage in Patients with Anti-GAD Antibodies (José Fidel Baizabal-Carvallo)

The clinical course of LACA is largely unknown. Emerging evidence suggests that patients with LACA may present with systemic or organ-specific autoimmune disorders before the onset of progressive ataxia, including neurological and systemic manifestations (Table 5). Moreover, a prodromal stage characterized by nonspecific symptoms including malaise, fatigue, or even cognitive complaints may be seen before the onset of neurological symptoms.

**Table 5:** Summary of clinical manifestations preceding or accompanying the onset of LACA

Systemic or organ-specific autoimmune disorder	
Latent autoimmune diabetes mellitus Overt type 1 diabetes mellitus Polyglandular syndrome type II (Schmidt syndrome) Autoimmune thyroiditis Vitiligo Pernicious anemia Myasthenia gravis Gluten sensitivity	
Neurological	
*Oculomotor* Horizontal nystagmus Downbeat nystagmus Upbeat nystagmus Multidirectional nystagmus Oculomotor paresis Opsoclonus Abnormal smooth pursuit Increased latency of saccades Decreased velocity of saccades	
*Other* Fluctuating vertigo Fluctuating limb or gait ataxia Axial stiffness Limb stiffness Epileptic seizures	

*Abbreviations*: *LACA*, latent autoimmune cerebellar syndrome

Oculomotor manifestations may precede the development of overt ataxia. Multidirectional, horizontal, upbeat, and downbeat nystagmus may present in patients with low-titer anti-GAD Ab [28, 66, 67]. These patients should be evaluated carefully as they may initially show relatively minor signs of ataxia, such as difficulties with tandem gait or mild dysmetria. Such relatively subtle cerebellar manifestations may progress with time, leading to overt CA [68].

A clinical picture with more diffuse brainstem manifestations may also be a presentation of LACA [11]. Emerging evidence suggests that about 25% and 35% of patients with ataxia and high anti-GAD Ab develop episodes of transient neurological dysfunction involving brainstem nuclei and cerebellar connections [11]. These so-called brainstem attacks manifest with horizontal and/or vertical diplopia, nystagmus, vertigo, nausea, vomiting, dysarthria, paralysis of the posterior pharyngeal wall, gait, and limb ataxia [11, 31, 34]. These prodromal symptoms may last from several minutes to days but even weeks or months; their frequency may also be extremely variable, and the episodes may be isolated or with a paroxysmal character. Although these episodes usually resolve spontaneously and patients initially do not present with evident CA, an insidious progressive cerebellar syndrome usually appears within the following 3 months, but longer latencies of up to 2 years have also been reported [11, 31]. The reversibility of such symptoms suggests a transient autoimmune impairment apart from conspicuous neuronal loss that will eventually lead to progressive cerebellar damage.

Besides brainstem manifestations, axial or appendicular muscle rigidity, sometimes with superimposed muscle spasms may antedate the onset of a progressive CA [31, 69]. Such manifestations are consistent with the “classic” or “focal/segmental” subtypes of SPS antedating the cerebellar syndrome, sometimes underlying, by years [33, 70].

The coexistence of systemic or organ-specific autoimmune disorders [11, 35] with LACA suggests the presence of an active multi-organ autoimmune response with different degrees of active antibody production. Low and high-titer anti-GAD Abs may be identified in patients with gluten ataxia, which is a form of sporadic ataxia associated with anti-gliadin and anti-transglutaminase 6 Abs [71]. Forty percent of patients with gluten ataxia were anti-GAD Ab positive with a mean titer of 25 U/mL [71]. Whether such low-titers of anti-GAD Abs contributed to the cerebellar damage is difficult to define; however, the titer of anti-GAD Abs decreased in a third of these patients following a gluten-free diet, a phenomenon related to clinical improvement [71].

Similarly, 70% of patients with anti-GAD ataxia were found to have positive serology for gluten sensitivity, some of which responded well to a gluten-free diet without requiring immunosupression. This suggests a significant overlap between gluten ataxia and anti-GAD ataxia [72]. Current literature does not associate LACA with underlying neoplasia; however, few patients with CA and high-titer of anti-GAD Abs have been found with occult neoplasia and may exceptionally exists with low anti-GAD Ab titer [31, 33, 66].

The morphological appearance of the cerebellum assessed by neuroimaging seems a poor predictor of anti-GAD Ab titer, as up to 43% of patients with high Ab titer do not have cerebellar atrophy on MRI [73].

In patients with high titer anti-GAD antibodies, improvement of oculomotor abnormalities may follow treatment with IVIg, corticosteroids, or plasma exchange; however, the response may be incomplete or selective to specific form of ocular motor deficits. Cyclophosphamide may provide benefit in those who are refractory to treatment with IVIg, plasma exchange, or steroids [66]. However, it is unclear what proportion of these patients will eventually evolve to overt CA with high-titer anti-GAD Ab. Miller Fisher syndrome with negative anti-GQ1b and relatively low anti-GAD Ab (<2000 IU/mL) has been reported to improve clinically with IVIg alongside with a decrease of anti-GAD Ab titer [74].

Despite the low-titer of anti-GAD Abs, improvement with monthly courses of IVIg has been registered in selected patients coupled with improvement in cerebellar perfusion [75, 76]. Intravenous methylprednisolone 1000 mg/day for 5 days followed by oral prednisone for 2 months or oral corticosteroids alone may also provide clinical benefit on different ataxia scales [28, 29].

In summary, the brainstem manifestations either as transient deficits, i.e., “brainstem attacks” or as oculomotor dysfunction are among the most notable preceding symptoms in patients with ataxia associated with high-titer and low-titer anti-GAD Abs. SPS-like, systemic, or organ-specific autoimmune disease may also be present. Immunotherapy usually relates with good outcomes and clinical improvement associates with decrease in anti-GAD Ab titer, even if they are low at baseline. Whether this is explained by publication bias through successfully treated patients should be clarified in further studies.

### Prodromal Stage in PCD (Sergio Muñiz-Castrillo, Alberto Vogrig, Bastien Joubert, Jérôme Honnorat)

PCD may be accompanied by extracerebellar involvement, the frequency, and severity of which largely depends on the associated antibody. In general, patients with anti-Yo Abs and those with anti-Tr/DNER Abs tend to manifest PCD as isolated or predominant neurological manifestation [51]. Nevertheless, a thorough neurological examination in patients with anti-Yo Abs can disclose mild signs or symptoms suggesting involvement of corticospinal tract (30–58%) [62, 77], brainstem disorders or cranial neuropathies (13%) [77], peripheral disorders ranging from hyporeflexia and mild sensory disturbances (54%) [62] to a clear diagnosis of peripheral neuropathy (10%) [77], or even gastrointestinal dysmotility in rare circumstances [77]. Sometimes, the cerebellar syndrome is so severe that it prevents the optimal mental status evaluation. Robust dysarthria and motor dysfunction interfere with the evaluation of the cognitive impairment. Despite these difficulties in the examination, approximately one-fifth of patients with anti-Yo Abs has clinical evidence of cognitive dysfunction, typically in the form of emotional lability and memory deficits [62]. It remains unclear if these deficits relate to the cognitive functions of the cerebellum (Schmahmann syndrome) or reflect the involvement of extra-cerebellar structures [78]. In a series of 28 patients with anti-Tr/DNER Abs, all but one patient had cerebellar involvement, which was isolated except for 2 cases (7%) who showed encephalopathy and sensory neuropathy [53].

PCD patients with other anti-neural Abs show distinctive associations with both central and peripheral neurological disorders (Table 3). Importantly, the extracerebellar involvement can be a clue for the associated antibody. For example, sensory neuronopathy with or without encephalomyelitis is typically associated with anti-Hu Abs [55], hearing loss and/or tinnitus is frequently associated with anti-KLHL11 Abs [59], opsoclonus-myoclonus in adults typically associate with anti-Ri antibodies [56, 57], while narcolepsy-cataplexy and hypopituitarism are common in patients with anti-Ma2 Abs [58].

Notably, these extra-cerebellar symptoms are sometimes subtle, and precede the manifestations of CA.

PCD is sometimes preceded by prodromal clinical symptoms such as nausea, vomiting, dizziness, and vertigo [79, 80]. In addition, some patients with anti-Ri Abs may present with an isolated action tremor that evolves into an overt PCD late in the disease [57]. Similarly, hearing loss or tinnitus antedate the development of cerebellar/brainstem dysfunction in nearly 25% of the patients with anti-KLHL11 Abs [59]. We observed that up to 37% of patients with ataxia and anti-KLHL11 Abs experienced transient, paroxysmal, episodes of vertigo, unbalance, and vomiting, which lasted from months to years prior to the development of a permanent cerebellar dysfunction [81].

Furthermore, the genetic abnormalities observed in ovary cancer of patients with anti-Yo PCD and leading to the immune breakdown responsible of PCD immune activation are present in some women many years before the development of the cerebellar symptoms [82]. These data suggest that in some patients the cerebellar immune reaction can be present many months and years before the clinical symptoms.

In conclusion, even in patients with PCD, many arguments suggest that the immune reaction is present in the cerebellum many weeks or months before the development of clinical symptoms suggesting that the concept of LACA can be extended to patients with PCD.

### Prodromal Stage in Gluten Ataxia (Marios Hadjivassiliou)

GA refers to an IMCA triggered by the ingestion of gluten in gluten-sensitive individuals [83, 84]. By definition, such patients will have CA in the presence of serological markers of gluten sensitivity (one or more of antigliadin, TG2, and TG6 antibodies) [85]. The presence of enteropathy defines coeliac disease (CD) but it is not a prerequisite for the diagnosis of GA.

It has been shown that up to 47% of patients with newly diagnosed CD, presenting to the gastroenterologists have abnormal MR spectroscopy of the cerebellum [86]. Clinical evaluation showed that 29% of the patients had evidence of mild gait ataxia and 11% had nystagmus. None of these patients, however, had been referred to or seen by a neurologist even if on direct questioning 24% reported some gait instability. It could be argued that patients with LACA for whom the diagnosis of CD is based primarily on gastrointestinal symptoms, early treatment and good outcome can be expected given the retained cerebellar reserve.

In our experience at the Sheffield Ataxia Centre, we often get referrals of patients who have had brain imaging for various reasons (e.g., headache) who are then noted to have evidence of cerebellar atrophy. We have a cohort of such patients with positive serology for gluten sensitivity who on clinical examination have no evidence of any detectable ataxia. Often these patients have abnormal spectroscopy of the cerebellum that improves with the introduction of gluten-free diet. These examples represent LACA where the cerebellar reserve is sufficient to compensate for the cerebellar atrophy and the clinical intervention with gluten-free diet can result in complete recovery.

As such, the use of gluten sensitivity-related antibodies and in particular antigliadin and TG6 Abs may be a useful biomarker of some cases of LACA. Equally the use of MR spectroscopy of the cerebellum may identify patients with reduced NAA/Cr in the cerebellar vermis and/or hemisphere implying cerebellar dysfunction without overt ataxia. A prospective evaluation of healthy volunteers who have serological evidence of gluten sensitivity may be helpful in better understanding LACA in the context of gluten sensitivity.

### Prodromal Stage in Post-infectious Cerebellar Syndrome (PiCS) (José Fidel Baizabal-Carvallo)

Post-infectious cerebellar syndrome (PiCS) is defined as acute cerebellar inflammation induced by immune-mediated mechanisms triggered by a bacterial or viral pathogen, such as mycoplasma pneumoniae, Epstein-Barr virus, varicella zoster virus, cytomegalovirus, Coxsackie B3, or following vaccination [6, 87, 88]. More recently, SARS-CoV2, the cause of COVID19, has been identified as a potential trigger [89, 90]. However, in a substantial proportion of these patients, there is no clear underlying or previous infection. Although this condition is more commonly observed in children, there are reports of adult cases as well [91]. PiCS should be readily differentiated from acute infectious cerebellitis, the latter being caused by direct cerebellar tissue invasion by a specific pathogen.

Antibodies directed against the glutamate receptor delta 2 (anti-GluRδ2) which is highly expressed in Purkinje cells have been identified in the serum and CSF of some patients with cerebellitis following vaccination and diverse infections [92–94]. Patients have a variable clinical course ranging from a self-limiting to a fulminant course leading to severe cerebellar damage or death in some instances [95]. MRI may show unilateral or bilateral T2-weighted hyperintensities in the cerebellum [96].

Molecular mimicry is presumed as the pathogenic mechanism, similar to Guillain-Barré syndrome or Sydenham chorea in rheumatic fever [97, 98]. It is unclear how the LACA hypothesis applies to PiCS, owing to the following considerations: (*1*) the disorder has a self-limited course, (*2*) the latency between infection and onset of neurological manifestations may be unclear, although it is considered to be short, usually lasting less than 10 days, and (*3*) prodromal symptoms may be related to the immunological response to the infection. However, the presence of fever, malaise, drowsiness, headache, nausea, vomiting, photophobia, or vertigo, once the infection has seemingly resolved [87, 99], suggests the presence of a smoldering inflammatory process that will eventually reach a threshold for CA.

## Biomarkers During Progression of LACA

The signs related to the prodromal stage in IMCAs need to be identified for early interventions. First, we examine how eye movements can be objective markers of prodromal phase prior to onset of immune-mediated CA (section “Eye Movement Abnormalities as Physiological Biomarker (Aasef G. Shaikh)”). In this section, we will further identify nature of movement deficits that can suggest prodromal phase of CA induced by diverse etiologies. Paroxysmal deficits that are at the epicenter of the prodromal phase in CA are discussed. Second, we also discuss possible autoimmune biomarkers for LACA (section “Dynamic Changes of Autoimmune Biomarkers During Progression of LACA (Christiane S Hampe)”). Dynamic changes in some cytokines might reflect the early immune-mediated insults to the cerebellum.

### Eye Movement Abnormalities as Physiological Biomarker (Aasef G. Shaikh)

#### Clinical Features of Subtle and Transient Ocular Motor Deficits

Ocular motor deficits are common in IMCA. They can be seen in its early prodromal phase, prior to onset of CA or gait impairment. The most common form of ocular motor deficit in the neurotology clinic is gaze-evoked nystagmus followed by downbeat nystagmus. Relatively rare deficits such as upbeat nystagmus, slow saccades, and opsoclonus are also reported. Traditional diagnostic algorithm of these deficits includes screening for autoantibodies and neuroimaging. The diagnostic workup frequently reveals subtle cerebellar atrophy affecting vermis or para-vermis cerebellar region. It is not uncommon for such deficits to have negative family history, genetic disorders, or any toxic exposure. In select cases, we may find low titers of anti-GAD Abs, or voltage-gated calcium channel Abs. Hence, it is likely that early, mild cases of dizzy patients, who were found to have downbeat nystagmus and/or gaze-evoked nystagmus with mild cerebellar atrophy have prodromal phase and LACA. In such cases, when the aforementioned Abs are detected, treatment with plasma exchange or IVIg has resulted in mixed responses; i.e., one patient had complete resolution of downbeat nystagmus, while in another case, the response was incomplete.

Paroxysmal ocular motor and vestibular deficits are not uncommon in LACA [31]. These patients have normal inter-ictal neurological examination. Their typical presentation is acute episodes of vertigo with background constant unsteadiness. Clinical examination may reveal provoked nystagmus, typically after hyperventilation. Occasionally, nystagmus is mild and only present in peculiar gaze orientation. Inter-ictal examination of ocular motor system reveals subtle deficits including curved trajectory of saccades, or mild dysmetria. Low-titer anti-GAD Abs are also associated with gravity independent upbeat nystagmus [67]. It is common to find heterogeneous gaze-holding deficit in the syndrome of anti-GAD Ab. These patients have waveform that has mixture of squarewaves, downbeat nystagmus, and opsoclonus. The opsoclonus superimposes upon the downbeat nystagmus, while robust squarewave jerks are present in the axis orthogonal to the downbeat nystagmus [100].

#### Mechanistic Underpinning of Paroxysmal or Transient Worsening of Ocular Motor Deficits in LACA

Transient ocular motor deficits in LACA can be due to reversible loss of motor control. Such deficits are typical of transient ischemia to the brainstem but can also present with early forms of neurodegenerative or immune disease when loss of cerebellar and brainstem function is not complete and there are residual tissues compensating for the damage that has already happened.

**Fig. 4:**
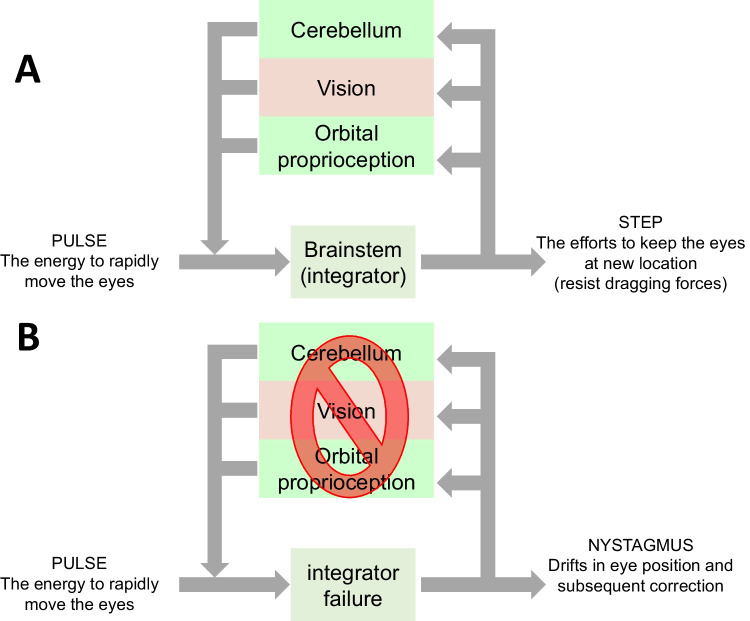
Schematic presentation of gaze holding network. The pulse of eye velocity signal is integrated by the brainstem neural integrators. This mechanism relies on normal function of the cerebellar Purkinje neurons, visual system, and orbital proprioception. As depicted in panel (**A**), the three sources of feedback, project to the input of brainstem neural integrators. As depicted in panel (**B**), the integration fails when one of the sources of feedback is impaired, either by damage of cerebellar Purkinje neurons, visual dysfunction, or disrupted orbital proprioception. The consequence of such abnormality is impaired neural integration. As a consequence, the eyes drift to the central null position, and drifts are followed by correction leading to phenomenology called “nystagmus.” The same sources of feedback, the cerebellum in particular, are also critical for assuring normal amplitude and directional matrix of saccade

Acute cerebellar inflammatory disorders present with robust cerebellar dysfunction in the acute phase, but treatment with immunomodulation or steroids results in a complete reversal of the abnormality. Ocular motor deficits can be sensitive markers of acute cerebellar dysfunction due to cerebellitis, acute immune reaction, or decompensated neurodegenerative disorders. One of the fundamental ocular motor behaviors included maintenance of the gaze requiring accurate utilization of the gaze-holding network and the brainstem neural integrator (Fig. 4). This network utilizes eye velocity signal from the saccade burst neurons and transforms it into gaze position signal by virtue of mathematical integration of the velocity signal. A fundamental limitation of the neural integrator is that it is always inaccurate. The inaccuracy is seen in the form of drift in eye position during eccentric gaze holding; the latter is typically compensated by the feedback from the visual system or cerebellar Purkinje neurons [101]. This task requiring accurately calibrated cerebellar signal is vulnerable to any form of cerebellar dysfunction. Any abnormality in the cerebellar outflow, either due to transiently inflamed Purkinje neurons sending aberrant output, or dead Purkinje neurons sending no output, leads to lack of calibration and dysfunction of neural integrator. The consequence is drifting eye position toward the central null position and eye-in-orbit position dependence of slow-phase eye velocity, the deficits that characterize the gaze-evoked nystagmus [101]. The gaze-evoked nystagmus is seen in patients with acute cerebellitis, acute autoimmune cerebellar deficit, and it can be treated with prompt medical management. In cases where damage to Purkinje cells is not complete, or it is mild, as seen in LACA, the deficits such as gaze-evoked nystagmus can be triggered by metabolically challenging the brain — hyperventilation-induced nystagmus is one of such examples. The patient with normal ocular motor examination at baseline may produce gaze-evoked or spontaneous nystagmus after 30-s-long hyperventilation.

The matrix of saccades, such as velocity and amplitude, is tightly controlled by the cerebellar Purkinje neurons. These cells provide critical error feedback and facilitate the enhanced accuracy of ongoing movements [101, 102]. Any impairment in the Purkinje neuronal function due to autoimmune or inflammatory disorders can impair the error feedback. Latter manifests in saccadic dysmetria. Reversal of saccade dysmetria is not uncommon after prompt treatment of autoimmune or inflammatory cerebellar conditions; however, permanent cerebellar damage results in irreversible saccadic dysmetria. Subtle saccade dysmetria is not unusual in compensated degenerative cerebellar disorders. Metabolic challenge due to acute medical illness can transiently affect cerebellar error control mechanism causing transient saccade dysmetria.

#### Mimics of Ocular Motor Deficits in Prodromal Phase of CA in LACA

Autoimmune or inflammatory deficits or prodromal phase of CA such as LACA are one way of transiently affecting the Purkinje neuron function. The other deficits include transient ischemia in the brain region supplied by the posterior circulation. These transient deficits do not present with diffusion abnormalities in the MRI, suggestive of acute stroke. Frequently, these deficits may resolve spontaneously without further intervention, and in some instances, aggressive hemodynamic management is warranted. The transient nature of brainstem attack due to vascular etiology has clear differences from that due to prodromal phase of CA due to immune or inflammatory etiologies. Immune or inflammatory etiologies can be seen in pediatric population, young adults, adults, and the elderly; in contrast, brainstem attack due to vascular etiology is typically accompanied by vascular risk factors and typically affects older adults. The duration of vascular brainstem attacks may span from several minutes or can last for days and may depend on the blood pressure. On the contrary, the brainstem attack due to inflammatory or immune etiology lasts from days to weeks; blood pressure has no correlation with symptoms. Nevertheless, vascular etiology should be seriously considered in differential to prodromal symptoms of LACA, because if untreated with appropriate anti-lipid and anti-platelet management transient ischemia to the posterior circulation can lead to permanent damage in form of cerebellar stroke.

Dysfunction of the ion channels determining the cell membrane properties can frequently lead to dysfunction of the cerebellar Purkinje neurons. These deficits can be seen in those with genetic channelopathy, leading to episodic ataxias, or intoxication from pharmacological substances, such as antiepileptics. The episodic ataxias (types EA2, EA3, and EA4) present with transient ocular motor dysfunction such as gaze-evoked nystagmus, positional nystagmus, saccade dysmetria, or acute vestibular dysfunction [103, 104]. The deficits can last from hours to days and are self-limiting. There is a familial trend, and there are minimal to no ocular motor dysfunction in the inter-ictal phase. Toxic increased levels of antiepileptics such as phenobarbital, fosphenytoin, lamotrigine, and carbamazepine are known to cause acute ocular motor cerebellar dysfunction and ataxia [105–112]. Typical features are downbeat nystagmus, gaze-evoked nystagmus, axial and appendicular ataxia, and gait instability. The deficits are transient and resolve with normalizing the antiepileptic levels. The mechanism of ocular motor dysfunction in those with toxic antiepileptic levels could be attributed to ion channel dysregulation. Channelopathy closely resembles immune etiologies and prodromal phase of LACA. They can present at any age; however, unlike immune etiologies, there is a family history. On the contrary, drug-induced vestibular and ocular motor symptoms correlate with increased serum concentration of the offending pharmacotherapeutic agent.

### Dynamic Changes of Autoimmune Biomarkers During Progression of LACA **(**Christiane S Hampe**)**

Latent autoimmune diseases such as LADA are characterized by a slowly progressing pathogenesis, which eventually results in irreversible tissue damage. The disease progression may be linear, or show a remission/relapse pattern, where periods of disease progression are followed by upregulation of anti-inflammatory immune responses, allowing partial recovery. Eventually, the tissue damage is too severe and restoration to normal function can no longer be achieved. The length of the prodromal period varies and may be impacted by genetic susceptibility, environmental factors, or diet. A clear understanding of the molecular events and timing involved in the final breakdown of the immune response is needed to develop intervention therapies. The dynamic nature of the pathogenesis is reflected in changing levels of immune factors, including cytokines and chemokines. In a recent publication [113], disease progression in LADA patients was found to correlate with a decline in levels of cytokines Interleukin-1 receptor agonist (IL-1ra) and interleukin-1 beta (IL-1b). IL-1ra is a receptor antagonist of IL-1 and its direct correlation with C-peptide levels in type 1 DM patients suggests an involvement of the anti-inflammatory cytokine in disease remission [114]. The concomitant decrease in pro-inflammatory cytokine IL-1 beta and anti-inflammatory cytokine IL-1ra in LADA patients demonstrates the complicated, interconnected system of cytokine regulation, where multiple factors are involved in the dynamic changes of cytokine release. While it remains to be determined whether the dynamic changes in cytokine pattern observed in LADA patients are cause or effect of the progressive decline in beta cell function, they may serve as novel biomarkers for disease progression. Further studies are necessary to establish whether changes in cytokine levels or other immune factors can be observed in LACA and can be used as biomarkers for disease progression.

## Conclusion (Manto M)

The concept of LACA has implications in terms of prevention and administration of early therapies. Like for LADA, a personalized approach is recommended. The ultimate goal is the preservation of the cerebellar reserve, both functional and structural. In ataxic patients with no manifestly evident autoimmunity, the significance of associated autoantibodies should be carefully assessed for diagnosis of LACA. Patients suspected to present LACA require a close clinical/biological/radiological follow-up with longitudinal observations. The annual progression rate is currently unknown and might be different at a very early stage and later during follow-up, as observed in genetic ataxias where a non-linear progression is found [115]. In preataxic SCA3 patients, elevated levels of neurofilament light (Nfl) are detected already 7.5 years before onset. There might be a similar window in IMCA [116]. The signs related to the prodromal stage need to be identified. IMCAs patients often report fatigue in the months before the ataxia onset. There is a need to obtain data related to cerebellar involvement and extra-cerebellar involvement. The inventory of non-ataxia signs (INAS total count) might be useful in IMCAs also. Regarding the cerebellar aspect, subtle ocular disturbances, motor deficits, and cognitive/affective symptoms need to be scrutinized and correlated with biomarkers.

## Data Availability

The concept reported in this manuscript is not associated with raw data.
